# Supplementary material for: Clinical characterization of Lassa fever: A systematic review of clinical reports and research to inform clinical trial design
Source: PLoS Negl Trop Dis. 2021 Sep 21;15(9):e0009788. doi: 10.1371/journal.pntd.0009788 (PMC8486098; doi:10.1371/journal.pntd.0009788)
Supplement: S1 Text — (DOCX) [file pntd.0009788.s001.docx]

**African Journal Online** (<https://www.ajol.info/>)

| Date: 02/12/2018  Search strategy:  --------------------------------------------------------------------------------  lassa* |
| --- |

**Cochrane Central Register of Controlled Trials (CENTRAL)** (<https://www.cochranelibrary.com/central/about-central>)

| Date: 07/01/2020  Issue 12 of 12, December 2019  Search strategy:  -----------------------------------------------------------------------------  #1 MeSH descriptor: [Lassa Fever] explode all trees 2  #2 MeSH descriptor: [Lassa virus] explode all trees 0  #3 lassa* 153  #4 #1 or #2 or #3 153 |
| --- |

**Embase** (<https://www.embase.com/login>)

| Database: Embase 1974 to present  Search strategy:  --------------------------------------------------------------------------------  1 Lassa fever/ (904)  2 Lassa virus/ (1000)  3 lassa*.ti,ab. (1554)  4 1 or 2 or 3 (1983)  5 4 (1983)  6 limit 5 to yr="1883 - 2019" (1978) |
| --- |

**Global Health (Ovid)** (<http://www.ovid.com/site/catalog/databases/30.jsp>)

| Database: Global Health <1973 to 2019 Week 51>  Search strategy:  --------------------------------------------------------------------------------  1 exp Lassa virus/ or exp Lassa fever/ (789)  2 lassa*.ti,ab. (812)  3 1 or 2 (851)  4 3 (851)  5 limit 4 to yr="1931 - 2019" (851) |
| --- |

**Global Index Medicus** <http://www.globalhealthlibrary.net/php/level.php?lang=en&component=17&item=107>

| Database: Global Index Medicus, regional indices only: African Index Medicus (AIM) and Index Medicus of the Eastern Mediterranean Region (IMEMR)  Search strategy:  -------------------------------------------------------------------------------  Lassa* |
| --- |

**PubMed/MEDLINE** (<https://www.nlm.nih.gov/bsd/pmresources.html>):

| Database: Medline (Ovid MEDLINE® Epub Ahead of Print, In-Process & Other Non-Indexed Citations, Ovid MEDLINE® Daily and Ovid MEDLINE®) 1946 to present  Search strategy:  --------------------------------------------------------------------------------  1 Lassa Fever/ (617)  2 Lassa virus/ (625)  3 lassa*.ti,ab. (1376)  4 1 or 2 or 3 (1512)  5 4 (1512)  6 limit 5 to yr="1860 - 2019" (1508) |
| --- |

**Clinicaltrials.gov**

| Date: 07/01/2020  -----------------------------------------------------------------------------  Condition or disease: Lassa – 10 |
| --- |

**ISRCTN Registry** (<https://www.isrctn.com/>editAdvancedSearch)

| Date: 07/01/2020  ------------------------------------------------------------------------  Condition: lassa – 0 |
| --- |

**Pan African Clinical Trials Registry** (<http://www.pactr.org/>)

| Date: 07/01/2020  Search Terms: Lassa – 0 |
| --- |

**WHO International Clinical Trials Registry** (<http://www.who.int/ictrp/en/>)

| Date: 21/9/2018  Advanced search: ALL trials  Condition: lassa – 69 |
| --- |
