# Supplementary material for: Clinical characterization of Lassa fever: A systematic review of clinical reports and research to inform clinical trial design
Source: PLoS Negl Trop Dis. 2021 Sep 21;15(9):e0009788. doi: 10.1371/journal.pntd.0009788 (PMC8486098; doi:10.1371/journal.pntd.0009788)
Supplement: S2 Text — (DOCX) [file pntd.0009788.s002.docx]

**Case study** - A study reporting observations on a single individual.

**Case series** - A study reporting observations on a series of individuals, with no control group

**Cohort study** - An observational study in which a defined group of people is followed over time.

**Case-control study** - A study that compares people with a specific disease or outcome of interest (cases) to people from the same population without that disease or outcome (controls).

**Cross-sectional study** - A study measuring the distribution of some characteristic(s) in a population at a particular point in time.

**Quasi-randomised study** - Methods of allocating people to a trial that are not random, but were intended to produce similar groups when used to allocate participants.
