# Supplementary material for: Clinical characterization of Lassa fever: A systematic review of clinical reports and research to inform clinical trial design
Source: PLoS Negl Trop Dis. 2021 Sep 21;15(9):e0009788. doi: 10.1371/journal.pntd.0009788 (PMC8486098; doi:10.1371/journal.pntd.0009788)
Supplement: S1 Table — (DOCX) [file pntd.0009788.s003.docx]

**Lassa Systematic Review Data Dictionary and Extraction Manual**

# STUDY

## Study identification and publication details

| **st_study_title** | Free text: Short Hand Reference (first author, publication year) and, *if applicable*, Scientific title of the study/trial, as stated at registration. E.g. ‘Botoni2007’ |
| --- | --- |
| **st_primary_pub** | Free text: Reference of the journal article or conference abstract, including all authors and full title. Formatting style: ‘Elsevier Harvard (with titles)’  *Author Surname, A., Year Published. Title. Publication Title Volume number, Pages Used. doi:DOI Number* |
| **st_pub_type** | Dropdown variable: describing the medium with which research results were reported  1, “**Full text publication**” \| 2, “**Conference abstract**” \| 3, “**Trial registry**” \| 4, “**Dissertation/thesis**” \| 5, “**Other**” |
| **st_pub_date** | Date of publication (**YYYY/MM/DD**) (earliest known, i.e. chose ‘ePub ahead of print’, ‘Published online’ date if available), or of presentation at a conference. If an exact publication date is unknown (online or published version), if possible, chose ‘manuscript accepted’ date and add 1 month; estimate it from the journal; or, arbitrarily DD=15 and MM=01 and year of publication. |
| **st_author** | Free text: name of the corresponding author |
| **st_author_institution** | Free text: name and country of the corresponding author’s institution |
| **st_author_contact** | Email address for the author or contact for correspondence |
| **st_oth_pub_no** | Number of additional publications (journal articles or conference abstracts) that cover the same population and/or provide supplementary information in addition to the primary study publication (i.e. protocol, subgroup analyses, sub-studies such as PK/PD results or follow-up results). If none enter “**0**”. |
| **st_oth_pub_1, 2, 3 etc** | Free text: Reference of the associated journal article or conference abstract, including all authors and full title. Formatting style: ‘Elsevier Harvard (with titles)’ |

## Cohort

### Number of cohorts within single study

| **st_num_cohort** | Number of cohorts within this single ‘study’ reported in this article/abstract (see Appendix for details). Is =1 for almost everything – unless >1 very distinct populations who are assessed/treated or described separately or are in 2+ different countries. Unless prospectively assigned interventional groups. (e.g. 3 countries = 3 cohorts; 2 groups of control and cases = 2 cohorts) |
| --- | --- |
| **st_coh_div_reas** | Checkbox variable: describing the Reason for the division of study cohorts: 1, “**Several countries**” \| 2, “**Different participant groups**” \| 3, “**Different protocols per group** ” (e.g. interventional trial of 2+ treatment regimens) |
| **st_coh_div_desc** | Free text: further justification for counting a single ‘study’ reported in this article/abstract as several ‘cohorts’ = set of participants sharing the same study meta-data characteristics (see Appendix for details). |

## Setting

### Where was the study conducted/cohort recruited?

### The number of participants included in each cohort.

| **Following variables completed for each cohort: 1,2,3 etc.** | |
| --- | --- |
| **st_coh1_country** | Dropdown variable: Name of the country of study. Country name based on UN classification: <http://unstats.un.org/unsd/methods/m49/m49regin.htm> |
| **st_coh1_endemic** | Dropdown variable: describing whether the site where the study was conducted/patients recruited is one of the 7 countries where Lassa is endemic (Benin, Ghana, Guinea, Liberia, Mali, Sierra Leone, Nigeria); “**Endemic**” or “**Non-Endemic**”. As per WHO: <https://www.who.int/en/news-room/fact-sheets/detail/lassa-fever> |
| **st_coh1_site** | Free text: most precise name and address of each and every site of recruitment, including longitude and latitude GPS coordinates, if available. Can be 2+ sites. |
| **st_coh1_region** | Dropdown variable: World region of the country of study. Use UN classification: <http://unstats.un.org/unsd/methods/m49/m49regin.htm> |
| **st_coh1_num_patients** | Integer: Total number of participants in the initial cohort; i.e. who were *INCLUDED* into the study *– if a clinical trial, include only those who received* the intended intervention (drug(s), control, placebo, no-treatment, etc.), with the intention to follow them up for outcome assessment. |

## Study Characteristics

### What study design was used. Were data collected prospectively or retrospectively?

### Was the study comparative/were multiple arms involved in the study?

| **st_design** | Dropdown variable: detailing the study type/design. Discrete choice:  1, “**RCT**” \| 2, “**Quasi-randomised trial**” \| 3, “**Cohort**” \| 4, “**Case-control**” \| 5, “**Case-report or series**” \| 6, “Cross-sectional study” \| 9, “**Other**”  *See Appendix for further description of categories* |
| --- | --- |
| **st_reg_id** | If [**st_design**] = “1” 🡪 **RCT** or “2” 🡪 **Quasi-randomised trial**  Free text: Trial registry identifier, if the trial was registered and registration could be found (i.e. mentioned in the publication, or identified through search in ClinicalTrials.gov, or WHO ICTRP registries). Enter “**-99**” if a registry ID is not found or unknown. Separate by semi colon if multiple registry identifiers  e.g. NCT9834808; ACT4576 |
| **st_datacol** | Dropdown variable: describing whether data collection for the study was performed prospective or retrospectively. Discrete choice:  0, “**Prospective**” \| 1, “**Retrospective**” |
| **st_comp** | Dropdown variable: describing whether the study was comparative. Were different participants exposed to different treatments/interventions/regimens? Were multiple study arms compared? Discrete choice: 0, “**No**” \| 1, “**Yes**” |

## When was the study conducted and for how long were patients followed-up?

| **st_start_date** | Start date of data collection/patient follow-up (**YYYY/MM/DD**). If the exact date is unknown, then, arbitrarily, DD=15 and MM=01. If the year of study start date is unknown, refer to [**st_end_date]** and estimate the start year by subtracting the trial and follow-up duration from the [**st_end_date]** year. |
| --- | --- |
| **st_end_date** | End date of data collection/patient follow-up (**YYYY/MM/DD**). If the exact date is unknown, if possible, estimate it from start date and duration of follow-up, or, arbitrarily DD=15 and MM=12. If the year of study end is unknown, arbitrarily select the year before the publication was submitted to or accepted for publication where provided or alternatively the year before the publication date. |
| **st_followup** | Patient follow-up duration in days. Longest observation period for the longest followed single patient. Consider up to the latest outcome measured. For calculations in days; assumptions 1 month=30 days, 1 year 365 days. |

## Research eligibility

| **st_eligibility** | Dropdown variable: Are there specific research inclusion/exclusion criteria specified? Discrete choice: 0, “**No**” \| 1, “**Yes**” |
| --- | --- |
| **st_inclusion** | If [**st_elegibility**] = 1, “**Yes**”  Free text: Description of the inclusion criteria. |
| **st_exclusion** | If [**st_elegibility**] = 1, “**Yes**”  Free text: Description of the exclusion criteria. |

# PATIENT POPULATION

### Include only data on Lassa patients in this section. E.g. if the study includes a Lassa-negative control group, do not include data on these patients.

### When did symptoms first start and what analyses were performed to diagnose Lassa? The number of participants with lab-confirmed lassa.

| **pa_symp_min** | Integer: Minimum number of days from onset of symptoms to study start date for any given patient |
| --- | --- |
| **pa_symp_max** | Integer: Maximum number of days from onset of symptoms to study start date for any given patient |
| **pa_conf_dx** | Dropdown variable: How was Lassa diagnosis confirmed in the patient population? Discrete choice:  0, “**No confirmation of diagnosis**” \| 1, “**Laboratory confirmed diagnosis**” \| 2, “**Clinically confirmed diagnosis**” \| 3, “**Mix of laboratory and clinically confirmed diagnosis**” \| 99, “**Unknown**”  *See Appendix for the definition of confirmed Lassa diagnosis* |
| **st_diag_meth** | If [**pa_conf_dx**] = **1 or 3**  Checkbox variable: describing the types of diagnostic tests used to confirm laboratory diagnosis of participants at baseline/study inclusion. Several options may apply:  1, “**RT-PCR**” \| 2, “**Antigen ELISA**” \| 3, “**IgM ELISA” \| 4, “IgG ELISA**” \| 5, “**Viral culture**” \| 6, “**ReLASV**” \| 7, “**IFA**” \| 8, “**IHC**” \| 9, “**Other**” \| 10, “**Compliment fixation**” \| 99, “**Unknown**”  *See Appendix for further description of categories* |
| **st_diag_perc** | Integer: Percentage of the reported cases that were laboratory confirmed (%) |

### What is the age range and gender split of included patients?

| **pa_age_min** | Minimum age of included participants, that is the youngest participant included in the study, in years. If minimum is not reported, use lower limit of eligible age range. If no eligible age range reported for adult participants, arbitrarily enter “**18**”. Validation changed from integer to number to accommodate decimal numbers (i.e. if age is given in months). |
| --- | --- |
| **pa_age_max** | Maximum age of included participants, that is the eldest participant included in the study, in years. If maximum is not reported, use upper limit of eligible age range. If no upper age range and no maximum age reported enter “**99**”. Validation changed from integer to number to accommodate decimal numbers (i.e. if age is given in months). |
| **pa_female** | Integer: Proportion of participants included in the study who are female (%). If not reported and a ratio of M:F is provided, calculate the number of females by applying the ratio to the total number of included participants. Enter “**-99**” if unknown. |
| **pa_male** | Integer: Proportion of participants included in the study who are male (5). If not reported and a ratio of M:F is provided, calculate the number of males by applying the ratio to the total number of included participants. Enter “**-99**” if unknown. |

### Are any patients described immunocompromised or pregnant?

| **me_preg_yn** | Dropdown variable: Are any pregnant women known to be included in the population? Discrete choice: 0, “**No**” \| 1, “**Yes**” |
| --- | --- |
| **me_hiv_yn** | Dropdown variable: Are any patients with HIV known to be included in the population? Discrete choice:  0, “**No**” \| 1, “**Yes**” |
| **me_imm_yn** | Dropdown variable: were immunocompromised patients (excluding HIV+) known to be included in the population? Discrete choice:  0, “**No**” \| 1, “**Yes**” |
| **pa_immuno_type** | If [**me_imm_yn]** = “1” 🡪 **Included**  Free text: type of immunocompromised condition (e.g. diabetes, tuberculosis, etc) |

### Clinical description at baseline (how do patients present)?

| **pa_clin_desc** | Checkbox variable: describing clinical symptoms observed at baseline/study inclusion. Several options may apply:  1, “**Fever**” \| 2, “**Sore throat**” \| 3, “**Vomiting**” \| 4, “**Weakness**” \| 5, “**Malaise**” \| 6, “**Headache**” \| 7, “**Diarrhoea**” \| 8, “**Cough**” \| 9, “**Abdominal pain**” \| 10, “**Chest pain**” \| 11, “**Muscle pain**” \| 12, “**Nausea**” \| 13, “**Bleeding**” \| 14, “**Hearing loss**” \| 15, “**Others**” \| 16, “**None**” \| 17, **“Pharyngitis”** \| 18, “**Tonsilitis**” \| 19, “**Conjunctivitis** \| 20, “**Facial oedema**” \| 21, “**Dizziness**” |
| --- | --- |
| **pa_clin_desc_fever** | If [**pa_clinical_desc**] = 1, “Fever”  Integer: Prevalence of fever among patients (%). Enter “-99” if unknown. |
| **pa_clin_desc_sore_throat** | If [**pa_clinical_desc**] = 2, “Sore throat”  Integer: Prevalence of sore throat among patients (%). Enter “-99” if unknown. |
| **pa_clin_desc_vomiting** | If [**pa_clinical_desc**] = 3, “Vomiting”  Integer: Prevalence of vomiting among patients (%). Enter “-99” if unknown. |
| **pa_clin_desc_weakness** | If [**pa_clinical_desc**] = 4, “Weakness”  Integer: Prevalence of weakness among patients (%). Enter “-99” if unknown. |
| **pa_clin_desc_malaise** | If [**pa_clinical_desc**] = 5, “Malaise”  Integer: Prevalence of malaise among patients (%). Enter “-99” if unknown. |
| **pa_clin_desc_headache** | If [**pa_clinical_desc**] = 6, “Headache”  Integer: Prevalence of headache among patients (%). Enter “-99” if unknown. |
| **pa_clin_desc_diarrhoea** | If [**pa_clinical_desc**] = 7, “Diarrhoea”  Integer: Prevalence of diarrhoea among patients (%). Enter “-99” if unknown. |
| **pa_clin_desc_cough** | If [**pa_clinical_desc**] = 8, “Cough”  Integer: Prevalence of cough among patients (%). Enter “-99” if unknown. |
| **pa_clin_desc_abdom_pain** | If [**pa_clinical_desc**] = 9, “Abdominal pain”  Integer: Prevalence of abdominal pain among patients (%). Enter “-99” if unknown. |
| **pa_clin_desc_chest_pain** | If [**pa_clinical_desc**] = 10, “Chest pain”  Integer: Prevalence of chest pain among patients (%). Enter “-99” if unknown. |
| **pa_clin_desc_muscle_pain** | If [**pa_clinical_desc**] = 11, “Muscle pain”  Integer: Prevalence of muscle pain among patients (%). Enter “-99” if unknown. |
| **pa_clin_desc_nausea** | If [**pa_clinical_desc**] = 12, “Nausea”  Integer: Prevalence of nausea among patients (%). Enter “-99” if unknown. |
| **pa_clin_desc_bleeding** | If [**pa_clinical_desc**] = 13, “Bleeding”  Free text: Prevalence of bleeding among patients (%). Enter “-99” if unknown. |
| **Pa-clin-desc-bleedsite** | If [**pa_clinical_desc**] = 13, “Bleeding”  List sites of bleeding, e.g. Epistaxis, haematemesis, haemoptysis, haematuria, gingival bleed, Conjunctival haemorrhage, skin, gums, duodenum, conjunctiva, nose, needle and cut-down sites, oral mucosa, gastrointestine, vagina. |
| **pa_clin_desc_hearing** | If [**pa_clinical_desc**] = 14, “Hearing loss”  Integer: Prevalence of hearing loss among patients (%). Enter “-99” if unknown. |
| **pa_clin_desc_pharyngitis** | If [**pa_clinical_desc**] = 17, “Pharngitis”  Integer: Prevalence of hearing loss among patients (%). Enter “-99” if unknown. |
| **pa_clin_desc_tonsilitis** | If [**pa_clinical_desc**] = 18, “Tonsilitis”  Integer: Prevalence of hearing loss among patients (%). Enter “-99” if unknown. |
| **pa_clin_desc_conjunctivitis** | If [**pa_clinical_desc**] = 19, “Conjunctivitis”  Integer: Prevalence of hearing loss among patients (%). Enter “-99” if unknown. |
| **pa_clin_desc_facialoedema** | If [**pa_clinical_desc**] = 20, “Facial oedema”  Integer: Prevalence of hearing loss among patients (%). Enter “-99” if unknown. |
| **pa_clin_desc_dizziness** | If [**pa_clinical_desc**] = 21, “Dizziness”  Integer: Prevalence of hearing loss among patients (%). Enter “-99” if unknown. |
| **pa_clin_desc_other** | If [**pa_clinical_desc**] = 15, “Others”  Free text: Description of the other clinical symptoms presented, with prevalence. Ex. Anorexia (22%), Dysuria (12%), Proteinuria (5%), Azotaemia (11%), Kalaemia (11%), Natremia (22%), Bacteriuria (28%) |
| **ou_labp** | Dropdown variable: Were any laboratory parameters assessed at baseline?  Discrete choice: 0, “**No**” \| 1, “**Yes**” |
| **pa_lab_measure** | If **[ou_labp]** = 1, “Yes”  Checkbox variable: What laboratory parameters were measured at baseline? Several options may apply: 1, “**Haematological**” \| 2, “**Liver function**” \| 3, “**Kidney function**” \| 4, “**Other**” \| 99, “**Unknown”** |
| **pa_lab_haem** | If [**pa_lab_ measure**] = 1, “**Haematological**”  Free text: Description of the test along with range and mean across all patients. Include units.  Pop-up instructions: Include Haemoglobin, WBC count, Neutrophils, Lymphocytes, Eosinophils, Monocytes, Basophils, Thrombocytes, Reticulocyte count, haematocrit/Packed Cell Volume, platelet count, Red blood cells, Erythrocyte sedimentation rate (ESR), Stab cells, Myelocytes, Juveniles Haemoglobin, and all clotting screens (prothrombin time, partial thromboplastin time, INR, bleeding time, coagulation time). |
| **pa_lab_ast** | If [**pa_lab_ measure**] = 2, “**Liver function**”  Free text: AST range (min & max). Include units. |
| **pa_lab_astmax** | If [**pa_lab_ measure**] = 2, “**Liver function**”  Free text: maximum AST mean across all patients. Include units. |
| **pa_lab_alt** | If [**pa_lab_ measure**] = 2, “**Liver function**”  Free text: ALT range (min & max). Include units. |
| **pa_lab_altmax** | If [**pa_lab_ measure**] = 2, “**Liver function**”  Free text: maximum ALT mean across all patients. Include units. |
| **pa_lab_hep_others** | If [**pa_lab_ measure**] = 2, “**Liver function**”  Free text: Description of any additional liver function tests reported at baseline, including range across all patients, with units.  Pop-up instructions: Include Serum bilirubin, Total bilirubin, Alkaline phosphatase, direct and indirect bilirubin, Thymol turbidity, Total serum protein, Albumin fraction, Serum cholesterol. |
| **pa_lab_ren** | If [**pa_lab_ measure**] = 3, “**Kidney function**”  Free text: Description of the test along with range and mean across all patients. Include units.  Pop-up instructions: Dip stick urinalysis (sodium, chloride, potassium, nitrites, protein, glucose, leukocytes), microscopic urinalysis (granular casts), albumin, blood urea nitrogen (BUN), blood urea, creatinine, serum creatinine, urine culture. |
| **pa_lab_other** | If [**pa_lab_ measure**] = 4, “**Other**”  Free text: Description of the test along with range and mean across all patients. Include units. |

# TREATMENTS

### Details of the intervention/treatment regimens (for the purpose of associating specific outcomes to specific treatments). Divisions of cohorts/arms/groups with respect to treatments is shown here by defining separate treatment regimens and the number of patients who had each.

| **Following variables completed for each treatment** | |
| --- | --- |
| **sa_num_int** | Integer: Number of interventions administered to patients – regardless of which arm. Include all interventions administered as research interventions, and all antiviral treatment or other pathogen-targeting treatment administered as non-research interventions as well as all symptomatic treatments and supportive care. |
| ***Branch logic*** *– following variables to be completed for each intervention administered - corresponding to* **[sa_num_intv]** | |
| **sa_intv1_target** | Dropdown variable: What type of intervention is this? One option only:  1, “**Antiviral treatment**” \| 2, “**Symptomatic treatment”** \| 3, “**Fluid and electrolyte management**” \|4, “**Other**” \|5, “**Other RCT intervention for Lassa treatment**” |
| **sa_intv1_desc** | Free text: Name of treatment(s) or intervention . |
| **sa_intv1_drug** | If **[sa_intv1_target]** = 1, “**Antiviral treatment**”  Checkbox variable: Name of drug administered / active pharmaceutical ingredient (API) as per INN. Can be both  1, “**Ribavirin**” \|2, “**Other**” |
| **sa_intv1_other** | If **sa_intv1_drug =** 2, Other: free text drug name(s) INN. *Controlled vocabulary: International Nonproprietary Names (INN) should be used to identify pharmaceutical substances or active pharmaceutical ingredients.* |
| **sa_intv1_dose** | Text: Dose administered if available (include units and timing) |
| **sa_intv1_dura** | Integer: Protocol specified (preferred) or longest duration in days of the length of treatment or days the intervention was administered since beginning. Enter “**-99**” if unknown. If duration of treatment varied, enter maximum number of days any included participant received the intervention. For calculations in days; assumptions 1 month=30 days, 1 year 365 days. |
| **sa_intv1_num** | Integer: Number of patients treated or exposed to the intervention. That is, the total number of Lassa patients in the study arm. Enter “**-99**” if unknown. |

# RCT ENDPOINTS

## Primary Treatment Related Outcomes

Were treatment/intervention related outcomes assessed at any time during the study post-baseline (during the treatment period or follow-up)?

### What is the primary research endpoint?

### What other endpoints does this study evaluate?

| **me_prim_end** | If [**st_design**] = “1” 🡪 **RCT** or “2” 🡪 **Quasi-randomised trial**  Free text: Primary endpoint - variable, time point, and how to measure  E.g. viral load – at day 28 – as measured by RT PCT  E.g. mortality – day 14 - |
| --- | --- |
| **me_num_end** | Dropdown list: 1, “**1**” \| 2, “**2**” \| 3, “**3**” \| 4, “**4**” \| 5, “**5**”  Number of secondary endpoint(s) |
| **me_sec_end1** | If [**me_num_end**] >= “**1**”  Free text – variable. Time point. How to measure.  E.g. viral load – at day 28 – as measured by RT PCR  E.g. mortality – day 14 - |
| **me_sec_end2** | If [**me_num_end**] >= “**2**”  Free text – variable. Time point. How to measure.  E.g. viral load – at day 28 – as measured by RT PCR  E.g. mortality – day 14 - |
| **me_sec_end3** | If [**me_num_end**] >= “**3**”  Free text – variable. Time point. How to measure.  E.g. viral load – at day 28 – as measured by RT PCR  E.g. mortality – day 14 - |
| **me_sec_end4** | If [**me_num_end**] >= “**4**”  Free text – variable. Time point. How to measure.  E.g. viral load – at day 28 – as measured by RT PCR  E.g. mortality – day 14 - |

# OUTCOMES

### What outcomes were assessed at any time during the study post-baseline (during the treatment period or follow-up)? Include only non-research-related outcomes, meaning those not captured by me_prim as above)

**Death**

### Were mortality-related outcomes assessed at any time during the study post-baseline (during the treatment period or follow-up)?

| **ou_mort** | Dropdown variable: Was mortality assessed at any time during the study post-baseline (during the treatment period or follow-up)?  Discrete choice: 0, “**No**” \| 1, “**Yes**” |
| --- | --- |
| **ou_mort_num** | If **[ou_ mort]** = 1, “Yes”  Integer: the case fatality rate (%) during the study follow-up. Enter “**-99**” if unknown. |
| **ou_mort_mean_length** | If **[ou_ mort]** = 1, “Yes”  Integer: The mean time in days from study inclusion to death.  Enter “**-99**” if unknown. For calculations in days; assumptions 1 month=30 days, 1 year 365 days. |

### Physiological or clinical

### Were clinical and laboratory parameters assessed at any time during the study post-baseline (during the treatment period or follow-up)?

| **pb_clin_desc** | Checkbox variable: describing the types of clinical symptoms post-baseline. Several options may apply:  1, “**Fever**” \| 2, “**Sore throat**” \| 3, “**Vomiting**” \| 4, “**Weakness**” \| 5, “**Malaise**” \| 6, “**Headache**” \| 7, “**Diarrhoea**” \| 8, “**Cough**” \| 9, “**Abdominal pain**” \| 10, “**Chest pain**” \| 11, “**Muscle pain**” \| 12, “**Nausea**” \| 13, “**Bleeding**” \| 14, “**Hearing loss**” \| 15, “**Others**” \| 16, “**Unknown**” \| 17, **“Pharyngitis”** \| 18, “**Tonsilitis**” \| 19, “**Conjunctivitis** \| 20, “**Facial oedema**” \| 21, “**Dizziness**” |
| --- | --- |
| **pb_clin_desc_fever** | If [**pb_clin_desc**] = 1, “Fever”  Integer: Prevalence of fever among patients (%).Enter “**-99**” if unknown. |
| **pb_clin_desc_sore_throat** | If [**pb_clin_desc**] = 2, “Sore throat”  Integer: Prevalence of sore throat among patients (%).Enter “**-99**” if unknown. |
| **pb_clin_desc_vomiting** | If [**pb_clin_desc**] = 3, “Vomiting”  Integer: Prevalence of vomiting among patients (%).Enter “**-99**” if unknown. |
| **pb_clin_desc_weakness** | If [**pb_clin_desc**] = 4, “Weakness”  Integer: Prevalence of weakness among patients (%).Enter “**-99**” if unknown. |
| **pb_clin_desc_malaise** | If [**pb_clin_desc**] = 5, “Malaise”  Integer: Prevalence of malaise among patients (%).Enter “**-99**” if unknown. |
| **pb_clin_desc_headache** | If [**pb_clin_desc**] = 6, “Headache”  Integer: Prevalence of headache among patients (%).Enter “**-99**” if unknown. |
| **pb_clin_desc_diarrhoea** | If [**pb_clin_desc**] = 7, “Diarrhoea”  Integer: Prevalence of diarrhoea among patients (%).Enter “**-99**” if unknown. |
| **pb_clin_desc_cough** | If [**pb_clin_desc**] = 8, “Cough”  Integer: Prevalence of cough among patients (%).Enter “**-99**” if unknown. |
| **pb_clin_desc_abdom_pain** | If [**pb_clin_desc**] = 9, “Abdominal pain”  Integer: Prevalence of abdominal pain among patients (%). Enter “**-99**” if unknown. |
| **pb_clin_desc_chest_pain** | If [**pb_clin_desc**] = 10, “Chest pain”  Integer: Prevalence of chest pain among patients (%).Enter “**-99**” if unknown. |
| **pb_clin_desc_muscle_pain** | If [**pb_clin_desc**] = 11, “Muscle pain”  Integer: Prevalence of muscle pain among patients (%).Enter “**-99**” if unknown. |
| **pb_clin_desc_nausea** | If [**pb_clin_desc**] = 12, “Nausea”  Integer: Prevalence of nausea among patients (%).Enter “**-99**” if unknown. |
| **pb_clin_desc_bleeding** | If [**pb_clin_desc**] = 13, “Bleeding”  Free text: Prevalence of bleeding among patients (%). Enter “**-99**” if unknown. |
| **Pa-clin-desc-bleedsite** | If [**pa_clinical_desc**] = 13, “Bleeding”  List sites of bleeding, e.g. Epistaxis, haematemesis, haemoptysis, haematuria, gingival bleed, Conjunctival haemorrhage, skin, gums, duodenum, conjunctiva, nose, needle and cut-down sites, oral mucosa, gastrointestine, vagina. |
| **pb_clin_desc_hearing** | If [**pb_clin_desc**] = 14, “Hearing loss”  Integer: Prevalence of hearing loss among patients (%).Enter “**-99**” if unknown. |
| **pa_clin_desc_pharyngitis** | If [**pa_clinical_desc**] = 17, “Pharngitis”  Integer: Prevalence of hearing loss among patients (%). Enter “-99” if unknown. |
| **pa_clin_desc_tonsilitis** | If [**pa_clinical_desc**] = 18, “Tonsilitis”  Integer: Prevalence of hearing loss among patients (%). Enter “-99” if unknown. |
| **pa_clin_desc_conjunctivitis** | If [**pa_clinical_desc**] = 19, “Conjunctivitis”  Integer: Prevalence of hearing loss among patients (%). Enter “-99” if unknown. |
| **pa_clin_desc_facialoedema** | If [**pa_clinical_desc**] = 20, “Facial oedema”  Integer: Prevalence of hearing loss among patients (%). Enter “-99” if unknown. |
| **pa_clin_desc_dizziness** | If [**pa_clinical_desc**] = 21, “Dizziness”  Integer: Prevalence of hearing loss among patients (%). Enter “-99” if unknown. |
| **pb_clin_desc_other** | If [**pb_clin_desc**] = 15, “Others”  Free text: Description of the other clinical symptoms presented, with prevalence. Ex. Anorexia (22%), Dysuria (12%), Proteinuria (5%), Azotaemia (11%), Kalaemia (11%), Natremia (22%), Bacteriuria (28%) |
| **ou_labp** | Dropdown variable: Were improvement of any laboratory parameters assessed at any time during the study post-baseline (during the treatment period or follow-up)?  Discrete choice: 0, “**No**” \| 1, “**Yes**” |
| **pb_lab_measure** | If **[ou_labp]** = 1, “Yes”  Checkbox variable: What laboratory parameters were measured during follow up? Several options may apply: 1, “**Haematological**” \| 2, “**Liver function**” \| 3, “**Kidney function**” \| 4, “**Other**” \| 99, “**Unknown”** |
| **pb_lab_haem** | If [**pb_lab_measure**] = 1, “**Haematological**”  Free text: Description of the test along with range and mean across all patients. Include units.  Pop-up instructions: Include Haemoglobin, WBC count, Neutrophils, Lymphocytes, Eosinophils, Monocytes, Basophils, Thrombocytes, Reticulocyte count, haematocrit/Packed Cell Volume, platelet count, Red blood cells, Erythrocyte sedimentation rate (ESR), Stab cells, Myelocytes, Juveniles Haemoglobin, and all clotting screens (prothrombin time, partial thromboplastin time, INR, bleeding time, coagulation time). |
| **pb_lab_ast** | If [**pb_lab_measure**] = 2, “**Liver function**”  Free text: AST range (min & max). Include units. |
| **pb_lab_astmax** | If [**pb_lab_measure**] = 2, “**Liver function**”  Free text: maximum AST mean across all patients. Include units. |
| **pb_lab_alt** | If [**pb_lab_measure**] = 2, “**Liver function**”  Free text: ALT range (min & max). Include units. |
| **pb_lab_altmax** | If [**pb_lab_measure**] = 2, “**Liver function**”  Free text: maximum ALT mean across all patients. Include units. |
| **pb_lab_hep_others** | If [**pb_lab_measure**] = 2, “**Liver function**”  Free text: Description of any additional liver function tests reported post-baseline, including range across all patients, with units.  Pop-up instructions: Include Serum bilirubin, Total bilirubin, Alkaline phosphatase, direct and indirect bilirubin, Thymol turbidity, Total serum protein, Albumin fraction, Serum cholesterol. |
| **pb_lab_ren** | If [**pb_lab_measure**] = 3, “**Kidney function**”  Free text: Description of the test along with range and mean across all patients. Include units.  Pop-up instructions: Dip stick urinalysis (sodium, chloride, potassium, nitrites, protein, glucose, leukocytes), microscopic urinalysis (granular casts), albumin, blood urea nitrogen (BUN), blood urea, creatinine, serum creatinine, urine culture. |
| **pb_lab_other** | If [**pb_lab_measure**] = 4, “**Other**”  Free text: Description of the test along with range and mean across all patients. Include units. |

## Pathogen Related Outcomes

### Were viral load related outcomes assessed at any time during the study post-baseline (during the treatment period or follow-up)?

| **ou_neg_ser** | Dropdown variable: Was negative seroconversion measured at any time during the study post-baseline (during the treatment period or follow-up)? I.e., Disappearance of antibodies (IgG/IgM ELISA, IFA). Discrete choice: 0, “**No**” \| 1, “**Yes**” |
| --- | --- |
| **ou_vir** | Dropdown variable: Was viral clearance assessed at any time during the study post-baseline (during the treatment period or follow-up)?  Discrete choice: 0, “**No**” \| 1, “**Yes**” |
| **ou_vir_measure** | If **[ou_vir]** = 1, “Yes”  Checkbox variable: How were follow-up viral load / clearance endpoints measured? Several options may apply:  1, “**RT-PCR**” \| 2, “**Antigen ELISA**” \| 3, “**IgM ELISA” \| 4, “IgG ELISA**” \| 5, “**Viral culture**” \| 6, “**ReLASV**” \| 7, “**IFA**” \| 8, “**IHC**” \| 9, “**Other**” \| 99, “**Unknown**” *See Appendix for further description of categories* |

## Life impact

### Were adverse events / side effects / safety variables / life impacts assessed any time during the study post-baseline (during the treatment period or follow-up)?

| **ou_comp** | Dropdown variable: Were other complications or disabilities assessed at any time during the study post-baseline (during the treatment period or follow-up)?  Discrete choice: 0, “**No**” \| 1, “**Yes**” |
| --- | --- |
| **ou_comp_def** | If **[ou_comp]** = 1, “Yes”  Free text: description of how complications were expressed (foetal mortality, deafness, life impact) |

# FURTHER DEFINITIONS

## Reasons for considering a single study as several cohorts

A study is to be divided accordingly into different cohorts if and only if at least one of the three following conditions apply:

1. The study clearly differentiates a priori between different categories of participants, such as infection by different strains → 1 cohort per participant group
2. Multi-centric study, with centres in several countries → 1 cohort per country
3. The protocol is otherwise amended depending on site/group (e.g. different diagnostic approach, different follow-up time-points) → 1 cohort per protocol

## Detailed explanation of study design categories

Definitions of study designs 1-5 are as per the Cochrane Consumers & Communication Review Group

Study Design Guide. For Review Authors. June 2013. <https://cccrg.cochrane.org/sites/cccrg.cochrane.org/files/public/uploads/Study_design_guide2013.pdf>

Definitions of study design 6, Diagnostic Test Accuracy is as per The Joanna Briggs Institute Reviewers’ Manual 2015. The systematic review of studies of diagnostic test accuracy. <https://joannabriggs.org/assets/docs/sumari/Reviewers-Manual_The-systematic-review-of-studies-of-diagnostic-test-accuracy.pdf>

1, “**RCT**”

RANDOMISED CONTROLLED TRIALS

In RCTs the investigator randomly assigns people to groups that will receive (intervention group) or not receive (control group) one or more interventions. The outcomes measured are then compared between the groups.

2, “**Quasi-randomised**”

QUASI-RANDOMISED CONTROLLED TRIALS

Trials that attempt to randomly assign participants to groups but use an inadequate approach to generate the random sequence are designated as quasi-randomised controlled trials. Such trials do attempt to randomly allocate participants with the intent of producing equivalent groups, but the randomisation methods used are not adequate because in practice they are relatively easy to manipulate or predict. Inadequate randomisation approaches: Alternation, Case record numbers, Birth dates, Week days or month of the year.

3, “**Cohort-study**”

COHORT STUDY (SYNONYM: FOLLOW-UP, INCIDENCE, LONGITUDINAL, PROSPECTIVE STUDY)

An observational study in which a defined group of people (the cohort) is followed over time. The outcomes of people in subsets of this cohort are compared, to examine for example people who were exposed or not exposed (or exposed at different levels) to a particular intervention or other factor of interest. A cohort can be assembled in the present and followed into the future (this would be a prospective study or a "concurrent cohort study"), or the cohort could be identified from past records and followed from the time of those records to the present (this would be a retrospective study or a "historical cohort study"). Because random allocation is not used, matching or statistical adjustment at the analysis stage must be used to minimise the influence of factors other than the intervention or factor of interest.

4, “**Case-control**”

CASE-CONTROL STUDY (SYNONYMS: CASE REFERENT STUDY, RETROSPECTIVE STUDY)

A study that starts with identification of people with the disease or outcome of interest (cases) and a suitable control group without the disease or outcome. The relationship of an attribute (intervention, exposure or risk factor) to the outcome of interest is examined by comparing the frequency or level of the attribute in the cases and controls. For example, to determine whether thalidomide caused birth defects, a group of children with birth defects (cases) could be compared to a group of children without birth defects (controls). The groups would then be compared with respect to the proportion exposed to thalidomide through their mothers taking the tablets. Case-control studies are sometimes described as being retrospective as they are always performed looking back in time.

5, “**Case-report or series**”

CASE STUDY (SYNONYMS: ANECDOTE, CASE HISTORY, SINGLE CASE REPORT)

An uncontrolled observational study involving an intervention and outcome for a single person (or other unit).

CASE SERIES

An uncontrolled observational study involving an intervention and outcome for more than one person.

6, “**Other**” Any other study design not captured in categories 1-7.

7, “**Cross-sectional study**”

CROSS-SECTIONAL STUDY (SYNONYM: PREVALENCE STUDY)

A study that examines the relationship between diseases (or other health related characteristics) and

other variables of interest as they exist in a defined population at one particular time. The temporal

sequence of cause and effect cannot necessarily be determined in a cross-sectional study.

## Definition of confirmed Lassa diagnosis

Definition of confirmed cases: Clinical signs and symptoms , laboratory confirmation (e.g. RT-PCR/ ELISA) and history (i.e. stay in endemic area or known foci of infection).

## Detailed explanation of diagnostic methods

1, “**RT-PCR**”

Reverse transcription polymerase chain reaction (PCR)

2, “**Antigen ELISA**”

ENZYME-LINKED IMMUNOSORBENT ASSAY (ELISA) is a method to detect antibodies against Lassa, or the antigen (Ag) directly.

3, “**IgM ELISA**”

ELISA assays capturing IgM antibodies

4, “**IgG ELISA**”

ELISA assays capturing IgG antibodies.

5, “**Viral culture**”

6, “**ReLASV**”

ReLASV® Pan-Lassa Antigen Rapid Test (Lassa Virus Nucleoprotein)

7, “**IFA**”

IMMUNOFLUORESCENCE ASSAY

8, “**IHC**”

Immunohistochemistry, performed on formalin-fixed tissue specimens, can be used to make a post-mortem diagnosis

9, “**Other**”

10, “**Compliment fixation**”

99, “**Unknown**”
