# Supplementary material for: Clinical characterization of Lassa fever: A systematic review of clinical reports and research to inform clinical trial design
Source: PLoS Negl Trop Dis. 2021 Sep 21;15(9):e0009788. doi: 10.1371/journal.pntd.0009788 (PMC8486098; doi:10.1371/journal.pntd.0009788)
Supplement: S2 Table — (DOCX) [file pntd.0009788.s004.docx]

| **Sign/symptom** | **Baseline** | | **Post-baseline** | |
| --- | --- | --- | --- | --- |
|  | **Individuals**  **n (%)** | **Publications**  **n (%)** | **Individuals**  **n (%)** | **Publications**  **n (%)** |
| Abdominal distension | - | - | 4/4 (100) | 4/147 (3) |
| Abdominal tenderness | 103/445 (23) | 2/147 (1) | - | - |
| Abdominal secretions | - | - | 1/1 (100) | 1/147 (1) |
| Abnormal posturing | - | - | 3/9 (33) | 1/147 (1) |
| Abnormal respiratory sounds | - | - | 18/42 (43) | 1/147 (1) |
| Absent reflexes | - | - | 1/1 (100) | 1/147 (1) |
| Aching legs | 1/2 (50) | 1/147 (1) | - | - |
| Adenopathy | 3/22 (14) | 1/147 (1) | 3/22 (14) | 1/147 (1) |
| Agitation | - | - | 1/1 (100) | 1/147 (1) |
| Albuminuria | - | - | 3/10 (30) | 1/147 (1) |
| Alopecia | - | - | 1/2 (50) | 1/147 (1) |
| Altered liver enzymes | - | - | 1/10 (10) | 1/147 (1) |
| Ankle clonus | - | - | 1/1 (100) | 1/147 (1) |
| Aphasia | 5/510 (1) | 1/147 (1) | 5/510 (1) | 1/147 (1) |
| Apnoea | - | - | 2/2 (100) | 2/147 (1) |
| Arthralgia | 15/30 (50) | 6/147 (4) | 87/445 (20) | 2/147 (1) |
| Arthritis | - | - | 4/42 (10) | 1/147 (1) |
| Ascites | 2/2 (100) | 2/147 (1) | 1/1 (100) | 1/147 (1) |
| Autophonia | 1/1 (100) | 1/147 (1) | 1/1 (100) | 1/147 (1) |
| Azotemia | - | - | 1/1 (100) | 1/147 (1) |
| Bacteriuria | - | - | 12/40 (30) | 1/147 (1) |
| Bitter taste | 8/198 (4) | 1/147 (1) | - | - |
| Bladder distension | 1/2 (50) | 1/147 (1) | - | - |
| Blindness | - | - | 2/31 (6) | 1/147 (1) |
| Blurred vision | - | - | 22/78 (28) | 2/147 (1) |
| Body pain | - | - | 6/46 (13) | 3/147 (2) |
| Bone pain | - | - | 1/2 (50) | 1/147 (1) |
| Bradycardia | - | - | 2/2 (100) | 2/147 (1) |
| Bradyphrenia | 1/1 (100) | 1/147 (1) | - | - |
| Breast pain | 2.03/7 (29) | 1/147 (1) | 15/37 (41) | 2/147 (1) |
| Catarrh | 1/2 (50) | 1/147 (1) |  |  |
| Change in smell | - | - | 7/47 (15) | 1/147 (1) |
| Chest infection | - | - | 1/1 (100) | 1/147 (1) |
| Chest pain | - | - | 1/1 (100) | 1/147 (1) |
| Cheyne-Stokes respiration | - | - | 1/1 (100) | 1/147 (1) |
| Chills | 18/25 (72) | 17/147 (12) | - | - |
| Chorioretinal scarring | - | - | 2/31 (6) | 1/147 (1) |
| Clenched jaw | - | - | 1/1 (100) | 1/147 (1) |
| Coated tongue | - | - | 2/10 (20) | 1/147 (1) |
| Conjunctival infection | 1/2 (50) | 1/147 (1) | - | - |
| Conjunctival pallor | 2/3 (67) | 2/147 (1) | - | - |
| Constipation | 1/1 (100) | 1/147 (1) | 16/42 (38) | 1/147 (1) |
| Crepitations | 2/7 (29) | 2/147 (1) | 2/6 (33) | 2/147 (1) |
| Cyanosis |  |  | 2/4 (50) | 2/147 (1) |
| Dark urine | 11/223 (5) | 4/147 (3) | - | - |
| Deafness | 1/7 (14) | 1/147 (1) | - | - |
| Dehydration | 3/19 (16) | 3/147 (2) | 5/45 (11) | 2/147 (1) |
| Depressed reflexes | - | - | 2/2 (100) | 2/147 (1) |
| Depression | - | - | 17/59 (29) | 3/147 (2) |
| Difficulty speaking | - | - | 9/47 (19) | 1/147 (1) |
| Difficulty swallowing | 5/11 (45) | 3/147 (2) | 28/88 (32) | 6/147 (4) |
| Difficulty walking | 2/2 (100) | 2/147 (1) | 28/49 (57) | 3/147 (2) |
| Dilated pupils | - | - | 1/1 (100) | 1/147 (1) |
| Distended jugular veins | 1/1 (100) | 1/147 (1) | - | - |
| Drowsiness | 1/1 (100) | 1/147 (1) | 1/1 (100) | 1/147 (1) |
| Dysgeusia | - | - | 18/47 (38) | 1/147 (1) |
| Dysmorphopsias | - | - | 1/2 (50) | 1/147 (1) |
| Dysphagia | 1/1 (100) | 1/147 (1) | 1/1 (100) | 1/147 (1) |
| Dysuria | 137/465 (29) | 4/147 (3) | 180/510 (35) | 4/147 (3) |
| Ear pain | 1/2 (50) | 1/147 (1) | 1/2 (50) | 1/147 (1) |
| Ecchymosis | - | - | 2/3 (67) | 2/147 (1) |
| Edema | 58/974 (6) | 4/147 (3) | 80/672 (12) | 4/147 (3) |
| Effusions | - | - | 4/42 (10) | 1/147 (1) |
| Emaciation | - | - | 1/1 (100) | 1/147 (1) |
| Encephalitis | 8.91/27 (33) | 1/147 (1) | - | - |
| Epididymitis | - | - | 2/3 (67) | 2/147 (1) |
| Exanthema | - | - | 4/42 (10) | 1/147 (1) |
| Exudative lesion of the tonsils | 1/1 (100) | 1/147 (1) | 1/1 (100) | 1/147 (1) |
| Eye pain | - | - | 2/31 (6) | 1/147 (1) |
| Eye redness | - | - | 2/31 (6) | 1/147 (1) |
| Facial weakness | - | - | 1/2 (50) | 1/147 (1) |
| Fainting | - | - | 2/47 (4) | 1/147 (1) |
| Flank pain | 1/1 (100) | 1/147 (1) | - | - |
| Flaring | - | - | 1/1 (100) | 1/147 (1) |
| Floaters | - | - | 1/31 (3) | 1/147 (1) |
| Flu-like illness | 3/9 (33) | 1/147 (1) | - | - |
| Flushing | 1/1 (100) | 1/147 (1) | - | - |
| Focal deficiency | 5.1/510 (1) | 1/147 (1) | 5/510 (1) | 1/147 (1) |
| Foetal death | - | - | 18/59 (31) | 2/147 (1) |
| Frequent micturition | 1/1 (100) | 1/147 (1) | - | - |
| Fullness | - | - | 1/1 (100) | 1/147 (1) |
| Gait anomaly | - | - | 5/22 (23) | 1/147 (1) |
| Glaucomatous optic neuropathy | - | - | 2/31 (6) | 1/147 (1) |
| Haematuria | 2/13 (15) | 1/147 (1) | - | - |
| Hair loss | - | - | 16/50 (32) | 2/147 (1) |
| Hallucinations | - | - | 8/47 (17) | 1/147 (1) |
| Head swelling | - | - | 11/44 (25) | 1/147 (1) |
| Hearing impairment | - | - | 1/1 (100) | 1/147 (1) |
| Heart failure | - | - | 20/291 (7) | 1/147 (1) |
| Hemolysis | - | - | 1/2 (50) | 1/147 (1) |
| Hepatomegaly | 14/49 (29) | 8/147 (5) | 13/71 (18) | 5/147 (3) |
| Hiccup | 5.1/510 (1) | 1/147 (1) | 22/934 (2) | 3/147 (2) |
| Hyperemic conjunctiva | - | - | 1/1 (100) | 1/147 (1) |
| Hyperglycaemia | 1/1 (100) | 1/147 (1) | - | - |
| Hypernatremia | - | - | 1/10 (10) | 1/147 (1) |
| Hypersalivation | 1/2 (50) | 1/147 (1) | - | - |
| Hypersensitivity | - | - | 4/42 (10) | 1/147 (1) |
| Hypertension | - | - | 21/292 (7) | 2/147 (1) |
| Hypoacusis | - | - | 1/2 (50) | 1/147 (1) |
| Hypokalemia | - | - | 1/10 (10) | 1/147 (1) |
| Hyponatremia | - | - | 1/1 (100) | 1/147 (1) |
| Hypopnea | - | - | 1/1 (100) | 1/147 (1) |
| Hypotension | 12/51 (24) | 4/147 (3) | 55/161 (34) | 13/147 (9) |
| Hypovolaemia | - | - | 1/2 (50) | 1/147 (1) |
| Hypoxemic respiratory failure | - | - | 1/1 (100) | 1/147 (1) |
| Hypoxia | - | - | 1/1 (100) | 1/147 (1) |
| Icterus | 1.1/22 (5) | 1/147 (1) | 9/22 (41) | 1/147 (1) |
| Impaired speech | 2/3 (67) | 2/147 (1) | - | - |
| Impaired vision | 5/510 (1) | 1/147 (1) | 5/510 (1) | 1/147 (1) |
| Incontinence | - | - | 5/43 (12) | 2/147 (1) |
| Inflamed palate | 1/1 (100) | 1/147 (1) | - | - |
| Insomnia | 1/1 (100) | 1/147 (1) | 1/2 (50) | 1/147 (1) |
| Iridocyclitis | 1/2 (50) | 1/147 (1) | 1/2 (50) | 1/147 (1) |
| Irregular pulse | 1/1 (100) | 1/147 (1) | - | - |
| Itching | - | - | 1/31 (3) | 1/147 (1) |
| Itching eyes | - | - | 4/31 (13) | 1/147 (1) |
| Jaundice | 32/537 (6) | 6/147 (4) | 33/632 (5) | 6/147 (4) |
| Laryngeal edema | - | - | 1/1 (100) | 1/147 (1) |
| Laryngeal stridor | - | - | 1/1 (100) | 1/147 (1) |
| Lassitude | - | - | 1/1 (100) | 1/147 (1) |
| Lattice degeneration | - | - | 1/31 (3) | 1/147 (1) |
| Lethargy | 1/1 (100) | 1/147 (1) | 3/5 (60) | 3/147 (2) |
| Leukopenia | - | - | 6/10 (60) | 1/147 (1) |
| Light-headedness | - | - | 26/49 (53) | 2/147 (1) |
| Limb pain | 1/1 (100) | 1/147 (1) | - | - |
| Liver failure | - | - | 1/1 (100) | 1/147 (1) |
| Liver tenderness | - | - | 2/3 (67) | 1/147 (1) |
| Loss of appetite | 1/1 (100) | 1/147 (1) | 2/2 (100) | 2/147 (1) |
| Loss of near vision | - | - | 1/31 (3) | 1/147 (1) |
| Low platelet levels | - | - | 1/10 (10) | 1/147 (1) |
| Low pulse pressure | 2/22 (9) | 1/147 (1) | 2/22 (9) | 1/147 (1) |
| Low urine output | - | - | 1/1 (100) | 1/147 (1) |
| Meningeal syndrome | 5/510 (1) | 1/147 (1) | 15/510 (3) | 1/147 (1) |
| Meningism | - | - | 4/42 (10) | 1/147 (1) |
| Meteorism | - | - | 4/42 (10) | 1/147 (1) |
| Moderately impaired visual acuity | - | - | 3/31 (10) | 1/147 (1) |
| Motivational anhedonia | 1/1 (100) | 1/147 (1) | - | - |
| Mouth lesions | - | - | 4/42 (10) | 1/147 (1) |
| Multiorgan systemic failure | - | - | 1/1 (100) | 1/147 (1) |
| Muscle cramps | - | - | 1/2 (50) | 1/147 (1) |
| Muscle spasms | - | - | 4/42 (10) | 1/147 (1) |
| Myelitis | 1/1 (100) | 1/147 (1) | - | - |
| Neck pain | 5/7 (71) | 2/147 (1) | 2/3 (67) | 2/147 (1) |
| Nerve deafness | - | - | 1/1 (100) | 1/147 (1) |
| Night sweats | 1/1 (100) | 1/147 (1) | 1/2 (50) | 1/147 (1) |
| Nuchal rigidity | - | - | 1/5 (20) | 1/147 (1) |
| Oliguria | 3/7 (43) | 1/147 (1) | 17/92 (18) | 5/147 (3) |
| Ophthalmodynia | - | - | 4/42 (10) | 1/147 (1) |
| Opisthotonos | - | - | 1/1 (100) | 1/147 (1) |
| Orchitis | - | - | 1/1 (100) | 1/147 (1) |
| Oral thrush | 1/2 (50) | 1/147 (1) | - | - |
| Pain (unspecified) | 34/79 (43) | 2/147 (1) | 33/57 (58) | 1/147 (1) |
| Pain in costal margin | - | - | 1/2 (50) | 1/147 (1) |
| Pale stool | 1/1 (100) | 1/147 (1) | - | - |
| Pallor | 6/14 (43) | 2/147 (1) | - | - |
| Pancreatitis | - | - | 1/2 (50) | 1/147 (1) |
| Papules | - | - | 2/2 (100) | 2/147 (1) |
| Paresthesia | - | - | 11/47 (23) | 1/147 (1) |
| Parotid swelling | 1/1 (100) | 1/147 (1) | 1/1 (100) | 1/147 (1) |
| Pericardial effusion | 1/1 (100) | 1/147 (1) | 3/34 (9) | 3/147 (2) |
| Pericarditis | - | - | 3/32 (9) | 1/147 (1) |
| Peripheral drusen | - | - | 1/31 (3) | 1/147 (1) |
| Peritonitis | - | - | 1/1 (100) | 1/147 (1) |
| Petechiae | 1/1 (100) | 1/147 (1) | 6/7 (86) | 5/147 (3) |
| Photophobia | 1/4 (25) | 1/147 (1) | 14/423 (3) | 1/147 (1) |
| Pleural adhesion | - | - | 1/1 (100) | 1/147 (1) |
| Pleural effusion | 3/66 (5) | 2/147 (1) | 11/84 (13) | 8/147 (5) |
| Pneumonia | 3/65 (5) | 1/147 (1) | 6/67 (9) | 2/147 (1) |
| Pneumonitis | - | - | 1/1 (100) | 1/147 (1) |
| Poor appetite | 1/13 (8) | 1/147 (1) | - | - |
| Polyuria | - | - | 4/42 (10) | 1/147 (1) |
| Positive kernig's sign | 4/4 (100) | 1/147 (1) | - | - |
| Pre-retinal haemorrhage | - | - | 1/31 (3) | 1/147 (1) |
| Preterm contractions | 2/7 (29) | 1/147 (1) | 10/37 (27) | 2/147 (1) |
| Prostration | 1/1 (100) | 1/147 (1) | 2/2 (100) | 2/147 (1) |
| Proteinuria | 2/13 (15) | 1/147 (1) | 257/442 (58) | 2/147 (1) |
| Pulmonary edema | - | - | 12/291 (4) | 1/147 (1) |
| Pulmonary embolism | - | - | 1/1 (100) | 1/147 (1) |
| Rales | 53/441 (12) | 1/147 (1) | 105/454 (23) | 3/147 (2) |
| Rash | 5/28 (18) | 5/147 (3) | 31/615 (5) | 8/147 (5) |
| Rash with erythema | 1/1 (100) | 1/147 (1) |  |  |
| Rectal prolapse | - | - | 4/42 (10) | 1/147 (1) |
| Renal angle tenderness | - | - | 10/31 (32) | 2/147 (1) |
| Renal dysfunction | - | - | 2/2 (100) | 1/147 (1) |
| Respiratory obstruction | - | - | 2/2 (100) | 2/147 (1) |
| Restlessness | 1/1 (100) | 1/147 (1) | 4/4 (100) | 4/147 (3) |
| Retinal fibrosis | - | - | 1/31 (3) | 1/147 (1) |
| Retrosternal pain | 5/8 (62.5) | 2/147 (1) | 79/116 (68) | 3/147 (2) |
| Rigor | 5/8 (62.5) | 4/147 (3) | 25/91 (27) | 2/147 (1) |
| Ronchi | - | - | 1/3 (33) | 1/147 (1) |
| Seizure | 14/520 (3) | 3/147 (2) | 41/592 (7) | 9/147 (6) |
| Semi-conscious | - | - | 1/1 (100) | 1/147 (1) |
| Septic shock | - | - | 5/40 (13) | 1/147 (1) |
| Septicaemia | - | - | 1/10 (10) | 1/147 (1) |
| Shallow breathing | - | - | 1/1 (100) | 1/147 (1) |
| Shoulder pain | - | - | 1/2 (50) | 1/147 (1) |
| Singultus | - | - | 4/42 (10) | 1/147 (1) |
| Skin desquamation | - | - | 4/42 (10) | 1/147 (1) |
| Skin tenderness | 1/1 (100) | 1/147 (1) | - | - |
| Somnolence | - | - | 1/1 (100) | 1/147 (1) |
| Sore mouth | - | - | 2/5 (40) | 1/147 (1) |
| Splenomegaly | 11/62 (18) | 4/147 (3) | 15/71 (21) | 5/147 (3) |
| Stiff neck | - | - | 1/1 (100) | 1/147 (1) |
| Stridor | - | - | 1/3 (33) | 1/147 (1) |
| Stupor | - | - | 1/1 (100) | 1/147 (1) |
| Substernal pleuritic pain | 1/1 (100) | 1/147 (1) | - | - |
| Suprapubic tenderness | 1/2 (50) | 1/147 (1) | 1/1 (100) | 1/147 (1) |
| Swollen neck and face | - | - | 1/1 (100) | 1/147 (1) |
| Syncope | 1/1 (100) | 1/147 (1) | - | - |
| Systolic murmur | 1/2 (50) | 1/147 (1) | - | - |
| Tachycardia | 3/22 (14) | 1/147 (1) | 5/25 (20) | 3/147 (2) |
| Tachypnoea | - | - | 6/44 (14) | 3/147 (2) |
| Tearing | - | - | 2/31 (6) | 1/147 (1) |
| Tenderness in right hypochondrium | 1/2 (50) | 1/147 (1) | - | - |
| Tension pneumothorax | - | - | 1/1 (100) | 1/147 (1) |
| Thickened bowel wall | - | - | 1/1 (100) | 1/147 (1) |
| Thrombocytosis | - | - | 1/5 (20) | 1/147 (1) |
| Tinnitus | 8/23 (35) | 2/147 (1) | 64/204 (31) | 9/147 (6) |
| Tonsillar exudates | - | - | 1/1 (100) | 1/147 (1) |
| Tremor | - | - | 27/56 (48) | 2/147 (1) |
| Ulcers | 5/30 (17) | 3/147 (2) | 7/23 (30) | 2/147 (1) |
| Unspecific neurological involvement | 112/300 (37) | 2/147 (1) | 78/181 (43) | 2/147 (1) |
| Unspecified chest symptoms | 1/1 (100) | 1/147 (1) | - | - |
| Unspecified urinary symptoms | 1/1 (100) | 1/147 (1) | - | - |
| Unsteadiness | - | - | 1/2 (50) | 1/147 (1) |
| Uremia | - | - | 1/10 (10) | 1/147 (1) |
| Vertigo | 10.56/22 (48) | 1/147 (1) | 29/71 (41) | 3/147 (2) |
| Vitreous opacity | - | - | 1/31 (3) | 1/147 (1) |
| Vocal cord paralysis | - | - | 1/1 (100) | 1/147 (1) |
| Weight loss | - | - | 27/47 (57) | 1/147 (1) |
| Wheeze | - | - | 1/3 (33) | 1/147 (1) |
| White patches on palate | 1/5 (20) | 1/147 (1) | - | - |
