# Supplementary material for: Clinical characterization of Lassa fever: A systematic review of clinical reports and research to inform clinical trial design
Source: PLoS Negl Trop Dis. 2021 Sep 21;15(9):e0009788. doi: 10.1371/journal.pntd.0009788 (PMC8486098; doi:10.1371/journal.pntd.0009788)
Supplement: S3 Table — (DOCX) [file pntd.0009788.s005.docx]

|  | **Baseline** | | **Post-baseline** | |
| --- | --- | --- | --- | --- |
| **Clinical sign** | **n/N articles  (%)** | **n/N patients (%)** | **n/N articles  (%)** | **n/N patients (%)** |
| **Death** | **-** | **-** | 109/147 (74) | 1896/6373 (30) |
| **Blood and lymphatic system disorders** | | | | |
| Lymphadenopathy^a^ | 8/147 (5) | 11/15 (73) | 14/147 (10) | 42/133 (32) |
| **Cardiac disorders** | | | | |
| Abnormal ECG^b^ | 1/147 (<1) | 1/22 (5) | 3/147 (2) | 51/56 (91) |
| **Ear and labyrinth disorders** | | | | |
| Hearing loss/ impairment* | 5/147 (3) | 14/734 (2) | 20/147 (14) | 80/1249 (6) |
| Tinnitus | 2/147 (1) | 8/67 (12) | 9/147 (6) | 64/204 (31) |
| Vertigo | 1/147 (<1) | 11/22 (50) | 3/147 (2) | 29/71 (41) |
| **Eye disorders** | | | | |
| Conjunctivitis* | 5/147 (3) | 131/736 (18) | 7/147 (5) | 233/977 (24) |
| **Gastrointestinal disorders** | | | | |
| Abdominal pain* | 32/147 (22) | 660/1581 (42) | 25/147 (17) | 1594/3039 (52) |
| Diarrhoea* | 33/147 (22) | 429/1612 (27) | 25/147 (17) | 1144/3120 (37) |
| Nausea* | 12/147 (8) | 24/43 (56) | 8/147 (5) | 462/1482 (31) |
| Pharyngitis* | 8/147 (5) | 232/449 (52) | 15/147 (10) | 361/531 (68) |
| Sore throat* | 29/147 (20) | 553/1604 (34) | 22/147 (15) | 661/1650 (40) |
| Tonsillitis* | 1/147 (1) | 1/2 (50) | 3/147 (2) | 3/4 (75) |
| Vomiting* | 28/147 (19) | 806/1631 (49) | 26/147 (18) | 1695/3077 (55) |
| **General disorders** | | | | |
| Bleeding* | 26/147 (18) | 334/1727 (19) | 45/147 (31) | 785/3387 (23) |
| Facial oedema* | 11/147 (7) | 83/1097 (8) | 14/147 (11) | 295/2480 (12) |
| Fever* | 81/147 (55) | 1527/1730 (88) | 51/147 (35) | 3067/3300 (93) |
| Malaise* | 17/147 (12) | 313/506 (62) | 11/147 (7) | 388/489 (79) |
| Weakness* | 18/147 (12) | 536/758 (71) | 13/147 (9) | 391/469 (83) |
| **Metabolic and endocrine disorders** | | | | |
| Anorexia | 8/147 (5) | 9/18 (50) | 10/147 (7) | 723/1829 (40) |
| **Musculoskeletal and connective tissue disorders** | | | | |
| Back pain | 9/147 (6) | 260/478 (54) | 7/147 (5) | 332/503 (66) |
| Joint pain | 8/147 (5) | 221/654 (34) | 5/147 (3) | 463/1833 (25) |
| Muscle pain* | 20/147 (14) | 259/998 (26) | 11/147 (7) | 826/2888 (29) |
| Non-specific chest pain* | 12/147 (8) | 509/1516 (34) | 15/147 (10) | 931/2812 (33) |
| **Nervous system disorders** | | | | |
| Dizziness* | 4/147 (3) | 403/957 (42) | 8/147 (6) | 506/1019 (50) |
| Encephalopathy | 1/147 (<1) | 9/27 (33) | 4/147 (3) | 59/333 (18) |
| Headache* | 40/147 (27) | 809/1622 (50) | 30/147 (20) | 2033/3200 (64) |
| Impaired consciousness^c^ | 11/147 (7) | 48/815 (6) | 34/147 (23) | 152/1667 (9) |
| Seizure | 2/147 (1) | 13/517 (3) | 8/147 (5) | 37/585 (6) |
| **Shock^d^** | **7/147 (5)** | **12/187 (6)** | **23/147 (16)** | **87/262 (33)** |
| Disseminated intravascular coagulopathy | 0/147 (0) | 0 | 1/147 (<1) | 1/1 (100) |
| Hypotension | 4/147 (3) | 12/51 (24) | 13/147 (8) | 87/262 (33) |
| Low pulse pressure | 1/147 (<1) | 2/22 (10) | 1/147 (<1) | 2/22 (10) |
| **Renal disorders** | | | | |
| Proteinuria | 1/147 (<1) | 2/13 (15) | 2/147 (1) | 257/442 (58) |
| Renal failure^e^ | 0/147 (0) | 0 | 6/147 (4) | 92/310 (30) |
| **Reproductive system and breast disorders** | | | | |
| Labour complications^f^ | 1/147 (<1) | 2/7 (29) | 4/147 (3) | 28/96 (29) |
| **Respiratory, thoracic and mediastinal disorders** | | | | |
| Breathing difficulty^g^ | 7/147 (5) | 21/310 (7) | 11/147 (7) | 265/1829 (14) |
| Cough* | 16/147 (11) | 556/1581 (35) | 21/147 (14) | 1235/3097 (40) |
| Hiccup | 1/147 (<1) | 5/510 (1) | 3/147 (2) | 22/934 (2) |
| Respiratory failure^h^ | 0/147 (0) | 0 | 3/147 (2) | 3/3 (100) |
| **Skin and subcutaneous tissue disorders** | | | | |
| Skin discolouration^i^ | 0/147 (0) | 0 | 7/147 (5) | 8/10 (80) |
| Skin rash^j^ | 6/147 (4) | 6/29 (21) | 12/147 (8) | 42/701 (6) |

*pre-identified signs and symptoms. All other signs and symptoms without an asterisk were considered clinically significant.

a Includes “Lymphadenopathy”, “Axillary lymphadenopathy”, “Cervical lymphadenopathy” and “Parotid lymphadenopathy”

^b^ Includes “Abnormal ECG”, “Arrythmia”, “Atrial fibrillation”, “Non-specific ST-T changes”, “ST-segment elevation”, “T-wave inversion”

^c^ Includes “Coma”, “Comatose”, “Unconsciousness”, “Syncope”, “Prostration”, “Lethargy”, “Confusion”, “Disorientation”, “Semiconsciousness”, “Agitation”, “Irritability”, “Clouded sensorium”, “Delirium”, “Hallucination”, “Irrational behaviour”, “Obtundity”, “Psychic alterations”

^d^ Includes totals of “Shock”, “Disseminated intravascular coagulopathy”, “Hypotension”, “Low pulse pressure”, “Vascular collapse”

^e^ Includes “Acute kidney injury”, “Renal failure”, “Renal dysfunction”

^f^ Includes “Foetal death”, “Pre-term contractions”

^g^ Includes “Breathing difficulty”, “Dyspnoea”, “Respiratory distress”, “Shortness of breath”

^h^ Includes “Respiratory failure”, “Hypoxemic respiratory failure”, “Hypoxia”

^i^ Includes “Ecchymosis”, “Petechiae”

^j^ “Erythema”, “Exanthema”, “Maculopapular rash”, “Papular eruptions on back”, “Rash”, “Rash with erythema”, “Skin desquamation”, “Skin rash”, “Urticaria”
