# Supplementary material for: Clinical characterization of Lassa fever: A systematic review of clinical reports and research to inform clinical trial design
Source: PLoS Negl Trop Dis. 2021 Sep 21;15(9):e0009788. doi: 10.1371/journal.pntd.0009788 (PMC8486098; doi:10.1371/journal.pntd.0009788)
Supplement: Attachment — Submitted filename: Response to Reviewer Comments 20AUG21.docx [file pntd.0009788.s007.docx]

20-Aug-2021

Dear Reviewers,

Thank you for providing useful suggestions for improvements of our manuscript. We sincerely appreciate the time you took to contribute to the improvement our work. A revised version of our manuscript has now been resubmitted that incorporates all changes made based on your comments.

Please see our response to each of your comments in the table below:

| **Reviewer comment** | **Author response** |
| --- | --- |
| The link between this review manuscript and how its data might be used to guide clinical trials needs to be better established. In the Introduction section, consider describing how other review papers guided clinical trials for treatments for other febrile illnesses | Thank you for this improvement. We have expanded the background to include examples of how this approach has been used to develop methodologies in cutaneous leishmaniasis and tuberculosis clinical trials. See lines 83-89 in the final version. |
| In the Discussion section consider adding a subsection describing how the results might be used to guide future ribavirin trials. Is there a proposed set of symptoms that should be considered for a suspected case definition? | In the discussion section titled ‘Case definition’ we have included information on how the results can inform the case definition of LF and how this can be applied to clinical trials. Expansion of this work will be detailed in a subsequent publication which specifically focuses on inclusion and exclusion criteria for clinical trials. See lines 325-332.    We have further specified within the background that our findings are targeted to LF trials in general, to include therapies beyond ribavirin. |
| While the focus of the study is to provide information regarding ribavirin trials, there is little information in the manuscript about selected studies that included ribavirin components. How many of the studies included ribavirin? Was ribavirin generally found to be effective in those studies? | We aim to develop a clinical trials framework for all clinical trials in LF. In response to this suggestion, we have clarified the generalisability of our approach in the background and discussion sections including the removal of a ribavirin specific focus. The discussion section on ‘Need for more clinical trials’ aims to further confirm this.    We have decided not to include details of ribavirin treatment included in the studies due to the low quality of reporting. 62% of 109 papers evaluated by JBI risk of bias assessment report exposure reliably and 68% of 139 studies report outcomes reliably. Furthermore, a comprehensive systematic review of ribavirin treatment in LF was recently published that includes most relevant papers from our review. <https://doi.org/10.1016/j.ijid.2019.07.015> We have explained this in lines 311-313. |
| The premise of this article seems to be that Lassa symptoms can be standardized across geographic regions. What are the implications of standardization? Different countries have different strains of LF. Also, public health units differ by time to clinical presentation and laboratory capacity (for evaluating lab-based items contributing to a suspected case definition). While it may be beyond the scope of this study to report in detail on those challenges, they do seem worth mentioning. Consider adding a subsection in the Discussion section about the challenges of standardization. | This was a particularly useful suggestion. We have taken this advice to rewrite the early parts of the discussion to now include a section on “Challenges and limitations of the current literature”. See lines 304 onwards. We have further included the outcome of a comparison of time to presentation across the three main countries in the results section. See lines 249-251. |
| Consider adding a subsection in the Discussion sections about the study limitations; e.g., test validity, little distinction between acute and long-term exposure. For instance, IgG testing measures long-term exposure and is associated with different symptoms than Ag+ acute cases | Thank you for this suggestion. We have used your feedback to expand our discussion on the limitations of the diagnostics used across the literature and the potential impact on the results presented. Please see the discussion section on ‘Diagnostic variation and confidence’ beginning at line 338. |
| The analysis follows a clear plan, the presentation of the result is clear and comprehensive. All figures and tables are of good quality. It is of note that the results include a broad range of clinical symptoms that go beyond what is often included in clinical case definitions. This corresponds to the numerous case reports that represent the broad clinical picture of LF and is thus an important contribution. The authors also recorded which clinical symptoms were more frequent after baseline.  The authors also analyzed the frequency of bleeding at different sites and found haematuria the most frequent presentation. **However, it should be more thoroughly explained which kinds of haemturia were documented, and if cases of microhaematuria were included, as it can be a result of kidney injury but does not necessarily represent true bleeding in the stricter sense to my opinion**.  Overall, the results are very informative in the effort of finding better standards for clinical studies on LF | Thank you for this supportive comment. We have reviewed all papers reporting haematuria. Only 2 of the papers, covering X and Y individuals at baseline and post-baseline respectively, report the type of haematuria. In both cases it is macroscopic haematuria. We have added this detail to the text to incorporate your suggestion. Please see lines 273-275. |
| The conclusions are sound in nature but it should be pointed out a little more that this analysis is strictly descriptive and that in order to reach the declared goal of establishing standardized protocols, results would need to be graded (for discussion). the rest of the discussion of the result is very well suited to inform the understanding of the topic. | This is a helpful comment. We have specified within the discussion that the analysis is strictly descriptive and that conclusions cannot be drawn from the results due to the low quality of evidence. We’ve clarified that the results of this analysis will be used only to ensure that the development of standardised data variables and outcomes reflects the entirety of the available evidence base. See lines 315-318. This is also reflected in the abstract and author’s summary. |
| Figure 3 needs to be graphically edited as the numbers are very hard to read, even on a large screen | Thank you. We have improved the quality of the figure. |
| Lines 12-13: "all studies were included"  This statement is overly broad as the literature search did not cover all available bibliographic databases and search engines (e.g., Google Scholar) | Thank you. We agree and have corrected this in a couple of places in the paper. |
| Line 17: what arose from these discussions? | Thank you for raising this need for clarification. We have amended this phrase in the abstract and the methods to explain that “A second reviewer verified all extracted data and resolved any discrepant data by reviewing the manuscript with a third reviewer.” |
| Lines 54-56. Further evidence in the form of clinical trials is required both to confirm the efficacy and safety of ribavirin and to test new therapies.  While this statement is true, consider elaborating on this statement by mentioning the advantages of testing in additional geographic regions, larger sample sizes, accounting for different LF strains, etc. | Thank you for this suggestion. We have built this into our discussion when addressing the need for new clinical trials and future work – and also made it one of our key conclusions. See lines 399-405. |
| Line 59-60: the clinical characteristics of LF and patient outcomes The second component of the sentence clinical characteristics of patient outcomes is confusing. Consider rewording. | Thank you for this suggestion. We have improved this phrasing to clarify our aims throughout the paper. |
| Line 87: spell out acronym for REDCap on first occurrence | Good point - done. |
| Lines 102-113: Overuse of “We” | Agreed – this has been addressed. |
| Line 225 (Table 5). The definition of laboratory confirmation is extremely influential, particularly at baseline. For instance, Ag ELISA measures acute exposure, IgM measures recent exposure, and IgG measures long-term exposure. It may be helpful to review Table 5 by diagnostic approach to determine whether pooling is justified. | You are right - thank you for this suggestion. We have used your feedback to expand our discussion on the limitations of the diagnostics used across the literature and the potential impact on the results presented. We’ve integrated evidence of reduced confidence of some diagnostics and colour-coded Table 5 to indicate those with greater and lesser confidence. See Table 5, and the discussion section on ‘Diagnostic variation and confidence’ beginning at line 338. |
| Line 262 – “An agreed”  Consider replacing agreed with uniform. | Agreed – now changed. |
| Lines 284-287: What are the reasons for this? Limited laboratory capacity? | Thank you for raising this. The lack of AST measurement and reporting is due to limitations in laboratory capacity and a lack of recognition of the importance of AST evaluation for safe ribavirin treatment. We have expanded the discussion section to detail why AST has been inadequately collected and reported in the historical literature and made suggestions on how this should be addressed. |
| Discussion section: seems to be missing any mention of the need for improved diagnostics and rapid testing | Good point. We have added a section on diagnostics to the discussion and included this point. Please see lines 353-354. |
| More data regarding Lassa virus epidemiology in the 'Introduction' section may further enhance the structural feature of the manuscript. The article " Systematic review and meta-analysis of the epidemiology of Lassa virus in humans, rodents and other mammals in sub-Saharan Africa. PLoS Negl Trop Dis. 2020 Aug 26;14(8):e0008589" may be useful to provide such information to the readers. | Thank you for the suggestion. This excellent paper has been incorporated to strengthen our background. |
| Line 61: I would suggest that the authors reword the purpose of the study. “individual-level data” is confusing with the reviews performed on individual patient data from the original databases of the authors of the included studies. This concept of individual data from included study participants should also be clarified throughout the manuscript. | Thank you for pointing this out. We have clarified this throughout the paper. |
| Line 99: The authors should clarify the detection methods and the targets (RNA, viral antigen, antibodies, IgM, IgG…) searched to be considered as confirmed cases of Lassa virus infection. | Thank you for this comment. We have clarified in the methods section that we have included “patients with a laboratory or clinically confirmed diagnosis of LF (including all diagnostic methods and studies that report patients with sequelae from a previous infection)”. See lines 102-104. As diagnostic criteria were not used as a search or selection criteria, we had no specificity on what type of diagnostics were used within the methods section - only our method for evaluating confidence in them. We report the types of diagnostics documented in the results section. |
| Line 102-106: In addition to the proportions reported, authors should ideally (if possible) provide the individual data of the included studies. | We have made all the primary data available as a supplementary file to this publication in excel format for ease of reuse. As more than half of the papers in the review are case reports, much of the data included in the dataset are individual-patient level data. Data from papers that report more than one case of LF are presented as aggregate data as per the publication. Due to the low quality of the data, and the challenges and investment required to access primary data, we did not see value in approaching the original authors to access further individual-patient level data. See Supplementary material 5. |
| The signs and symptoms reported in this work should be fully discussed in relation to the multiple other febrile illnesses and haemorrhagic fevers due to other pathogens present in these endemic areas of West Africa and presenting the same clinical picture. | We agree. We have expanded the discussion section to include this, strengthening the discussion on challenges of LF diagnosis. Please see the discussion section on ‘Case definition’, particularly lines 325-332. |
| The discussion needs more references to better inform the concept of the research. For example, line 291, what is the work that argues that pregnant women are at higher risk of death from infection with Lassa virus. | Thank you for this good suggestion. We have expanded the justification and references in the discussion as such, including evidence of heightened mortality in health care workers and pregnancy. We have also expanded this in the background section. Please see lines 290-295 and 49-51. |

Thank you for considering these changes we have made. We look forward to your decision.

Sincerely,

Josephine Bourner

Clinical Trials Manager

Centre for Tropical Medicine and Global Health, Nuffield Department of Medicine, University of Oxford
